# A coordinated network of ABA signaling, photorespiratory and proteostasis in EMS-induced maize mutant with enhanced drought resistance

**DOI:** 10.3389/fpls.2026.1828125

**Published:** 2026-06-12

**Authors:** Xiaodong Wang, Yantian Lu, Shenqi Gao, Yejian Wang, Xiaoqing Xie, Lei Zhang, Huaijun Tang, Cheng Liu

**Affiliations:** 1 Institute of Crops Research, Xinjiang Uygur Autonomous Region Academy of Agricultural Sciences, Urumqi, China; 2 Xinjiang Academy of Agricultural and Reclamation Sciences, Shihezi, China

**Keywords:** ABA signaling, CAT2, EMS, proteostasis, Zea mays

## Abstract

Water deficit poses a significant constraint on maize (*Zea mays* L.) productivity, underscoring the need to identify novel genetic resources with enhanced resilience. Here, an ethyl methanesulfonate (EMS)-induced maize mutant, *ems21S373204*, displayed an enhanced drought-tolerant phenotype featured by stay-green leaves and an increased survival rate. Physiological characterization indicated that this mutant maintains higher leaf water content and more stable membrane integrity under water deficit, suggesting favorable physiological adjustments associated with drought resistance. Comparative transcriptomic profiling indicated that this tolerance is mediated by the transcriptional upregulation of the biosynthetic gene 9-cis-epoxycarotenoid dioxygenases (N*CED6*), alongside the concurrent suppression of the negative regulator clade A type 2C protein phosphatases 9 (*PP2C9*). This signaling cascade is further linked to the activation of the antioxidant gene *CAT2*, leading to efficient scavenging of ROS and reduced lipid peroxidation. Crucially, metabolomic analysis uncovered a distinct metabolic reconfiguration in the mutant: contrasting with the stress-induced proteolysis observed in the wild type, the mutant exhibited preserved protein homeostasis, as evidenced by elevated soluble protein levels and suppressed accumulation of free amino acids. Furthermore, an integrated multi-omics analysis highlighted the concurrent upregulation of L-arginine:glycine amidinotransferase (*AGAT*) and Hypoxanthine Phosphoribosyltransferase (*HPR*) genes in conjunction with glycerate accumulation, suggesting that the photorespiratory pathway serves as an adaptive energy dissipation mechanism to safeguard the photosynthetic apparatus. These findings collectively elucidate a coordinated regulatory network in *ems21S373204* that prioritizes nucleotide homeostasis, metabolic flexibility, and cellular homeostasis over passive survival, offering a valuable genetic framework for breeding climate-resilient crops.

## 1 Introduction

Drought significantly limits global crop productivity and is expected to worsen with climate change. Maize (*Zea mays* L.), a key C4 cereal with high yield potential, contributes substantially to global food, feed, and industrial demands. Global reports show that more frequent and longer drought have already begun to depress yields of major cereals (FAO, 2024), with maize being among the most vulnerable because of its high water demand during rapid vegetative growth (Leng and Hall, 2019; Zhao et al., 2017). Moderate to severe drought can reduce maize yield by 15-60% in several production regions (Du et al., 2025; Santini et al., 2022; Sheoran et al., 2022). As warming and drying trends continue, developing drought-tolerant maize is crucial for sustainable agriculture and food security amidst more frequent extreme weather.

Over recent decades, extensive efforts have been made to understand drought tolerance in maize, focusing on physiological, biochemical, and genetic aspects. Physiologically, drought-tolerant maize shows adaptive traits like reduced leaf water loss and maintained membrane integrity, aided by antioxidant defenses that prevent oxidative damage (Cao et al., 2025; Niu et al., 2024; Sattar et al., 2022). Genetically, abscisic acid (ABA) plays a key role in drought response by influencing stomatal closure, root architecture, and stress-responsive gene expression (Muhammad Aslam et al., 2022; Xiong et al., 2002). In parallel, transcription factors such as DREB/CBF, NAC, bZIP, and MYB regulate protective pathways under water deficit (Cao et al., 2025; Kimotho et al., 2019; Lata and Prasad, 2011). Previous studies have demonstrated that photorespiration can alleviate oxidative damage caused by drought stress by reducing reactive oxygen species accumulation and maintaining the stability of the photosynthetic electron transport chain (Noctor and Foyer, 2002; Peterhansel et al., 2013). This protective mechanism complements the known drought adaptation pathways, further enriching the theoretical basis of plant drought tolerance research (Busch et al., 2020). Together, these studies shaped the current model that effective maize drought tolerance depends on the tight coupling of osmotic regulation, ROS scavenging and hormone-regulated transcriptional reprogramming.

With the advancement of QTL mapping, Genome-Wide Association Study (GWAS), and candidate gene cloning, numerous drought-related loci and genes have been identified in maize. However, their allelic diversity and context-dependent effects often limit their application in breeding (R et al., 2025; van Heerwaarden et al., 2012). Moreover, traditional breeding methods, which depend on existing natural variation and recurrent selection, face challenges in continuously identifying novel functional alleles, especially for complex quantitative traits such as drought tolerance. These traits are governed by intricate regulatory networks and are significantly influenced by developmental stages and environmental conditions (Cooper and Messina, 2023; Liu and Qin, 2021). Ethyl methanesulfonate (EMS) mutagenesis offers an efficient, cost-effective, and widely utilized strategy to induce a high density of point mutations, facilitating the generation of extensive allelic variants and populations (Chen et al., 2022). A gene-indexed maize EMS mutant collection has been developed, demonstrating that single-nucleotide variants responsible for altered agronomic or stress-related phenotypes can be rapidly identified (Lu et al., 2018). Unlike physical mutagens, EMS typically avoids large deletions, thereby simplifying downstream transcriptomic and metabolomic analyses (Chen et al., 2022). Importantly, Several studies on cereals have demonstrated that when a drought-tolerant line is an EMS mutant, the integration of transcriptomic and metabolomic data more effectively identifies mutation-responsive hubs compared to natural germplasm contrasts, due to reduced background noise (Arriagada et al., 2025; Y. Wang et al., 2025).

In this study, we identified an EMS-induced maize mutant with enhanced drought tolerance compared to its wild type. We assessed key drought-related traits to see if the mutant better maintained water status, membrane stability, and protein homeostasis during drought and recovery. We also conducted comparative transcriptome sequencing and untargeted metabolomics to identify drought-responsive changes due to the EMS mutation. Finally, we aimed to build a “gene–metabolite–phenotype” association framework to understand how EMS-induced nucleotide changes can alter stress-responsive networks in maize. Taken together, our working hypothesis is that the EMS mutation activate a specific drought-responsive regulatory module, which confers a coordinated improvement in water retention, membrane protection, and metabolic adjustment.

## 2 Materials and methods

### 2.1 Plant materials and growth conditions

The wild-type (WT) maize (Zea mays L.) utilized in this study was the Chang7 lines. Ethyl methanesulfonate (EMS) mutagenesis was conducted on dry seeds by treating them with 0.3-0.5% (v/v) EMS in a 0.1 M phosphate buffer (pH 7.0) under gentle shaking at ambient temperature for a duration of 12 hours. Treated seeds were thoroughly rinsed for 2 hours and subsequently dried prior to sowing. The M_1_ generation plants were self-pollinated to produce M_2_ families. Drought-tolerant phenotypes were identified at the seedling stage, leading to the selection of a stable drought-tolerant mutant line (Wang et al., 2025), designated as ems21S373204, for further phenotypic characterization and multi-omics analyses.

Seeds of WT and *ems21S373204* were surface-sterilized in 75% (v/v) ethanol for 3 min followed by 3% (w/v) sodium hypochlorite for 10 min, then rinsed several times with sterile water. Germination and subsequent growth were performed in plastic pots containing a sterilized mixture of peat soil and sand (1:1, v/v). Plants were maintained in a growth chamber under controlled conditions: light intensity of 300 µmol photons m^−2^ s^−1^ light, 75% humidity, a 30/25 °C day/night temperature, and a 12/12 h photoperiod.

### 2.2 Drought treatments and physiological analyses

A greenhouse with a 16 h light/8 h dark photoperiod at 28 °C was used for planting all materials. At the 3-leaf stage (about 21 days post-sowing), two genotypes (WT and *ems21S373204*) were split into normal watering and drought treatments, forming four groups: wild type with normal watering (NW), mutant with normal watering (NM), wild type under drought (DW), and mutant under drought (DM). In the drought stress treatment, mutant and wild-type plants were planted in pots. Each pot was filled with equal volumes of well-mixed soil, and 32 seeds were planted per line. A total of 2 L of water was evenly sprinkled, and after 20 days of water control, noticeable phenotypic differences were observed between the WT plant and the mutant plant. Drought was induced by stopping irrigation until soil moisture reached 30 ± 5% field capacity (FC) for about 8 days, then rewatered to 80-90% FC. During the treatment period, the soil moisture content measured by the gravimetric pot-weight method. Normal watering was maintained at 80-90% FC (Fatemeh et al., 2025). The experiment used a randomized block design with at least five biological replicates per group. For each group, tissue was harvested from the same leaf position, frozen in liquid nitrogen, and stored at −80 °C for further transcriptomics, metabolomics and omics assays analysis.

*Relative water loss rate (WLR)*: The youngest fully expanded leaf of WT and *ems21S373204* under normal condition was excised, weighed immediately (FW_0_), then air-dehydrated on the bench (25°C) and weighed at 1–8 h (FW_t_). For each time, WLR = (FW_0_-FW_t_)/FW_0_ × 100 (Perera-Castro et al., 2026). The samples in this section are from an independent processed sample to avoid interference from tissue resection with subsequent biochemical or molecular analysis.

*Relative water content (RWC)* was determined according to the method of Barrs & Weatherley (Barrs and Weatherley, 1962). Briefly, leaves of each group were sampled to obtain fresh weight (FW), floated on deionized water in the dark at 4 °C to obtain turgid weight (TW), then oven-dried to constant weight to obtain dry weight (DW). RWC (%) = (FW − DW)/(TW − DW) × 100.

*Relative electrolyte leakage (REL)* was measured following the approach described by Blum & Ebercon (Blum and Ebercon, 1981). Leaves were incubated in deionized water, initial conductivity (C_1_) was recorded after incubation. Samples were then boiled for 10 min, cooled to room temperature, and final conductivity (C_2_) measured. REL (%) = C_1_/C_2_ × 100.

*Malondialdehyde (MDA) content* was estimated by the thiobarbituric acid reactive substances assay with modifications as described previously (Dhindsa et al., 1981). Briefly, leaf tissue was homogenized in 5 mL 5% (w/v) trichloroacetic acid (TCA), centrifuged at 10, 000 × g for 10 min; 1 mL supernatant mixed with 4 mL 0.5% (w/v) thiobarbituric acid (TBA) in 20% TCA, heated at 95 °C for 15 min, then rapidly cooled on ice and centrifuged at 10, 000 × g for 10 min. Absorbance of the supernatant was read at 532 nm and 600 nm. Calculation of MDA concentration were performed according to the cited protocol.

*Total soluble protein (TSP)* was quantified using the Bradford method (Bradford, 1976). Absorbance was measured at 595 nm and results were expressed per unit fresh weight.

*Survival rate (SR)* was recorded 48 h after rewatering (2 days post-recovery). For each genotype, independent plants were recorded (n=10). Survival rate (%) = (number of surviving plants/total plants) × 100.

### 2.3 RNA extraction, library construction and transcriptomic sequencing

For transcriptomics, the fully expanded leaves of each group were sampled and frozen in liquid nitrogen and stored at −80 °C. Three independent biological replicates were performed for each group. Total RNA was extracted using TRIzol reagent following manufacturer’s instructions. RNA integrity was verified by Agilent Bioanalyzer 4150 system (Agilent Technologies, CA, USA) with RIN ≥ 7.0. Paired-end libraries were prepared using a ABclonal mRNA-seq Lib Prep Kit (ABclonal, China) following the manufacturer’s instructions and sequenced an Illumina Novaseq 6000 and 150 bp paired-end reads were generated. Sequencing and library construction were performed by Shanghai Applied Protein Technology.

Raw reads were processed with fastp (v0.18.0) (Chen et al., 2018) to remove adapters low-quality reads. Ribosomal RNA reads were removed by aligning to the rRNA database using Bowtie2 (Langmead and Salzberg, 2012) (v2.2.8). Clean reads were aligned to the reference genome (https://plants.ensembl.org/Zea_mays/Info/Index) using HISAT2 (v2.1.0) (Kim et al., 2015). FeatureCounts (http://subread.sourceforge.net/) was used to count the reads numbers mapped to each gene. And then FPKM of each gene was calculated based on the length of the gene and reads count mapped to this gene. The annotation of gene was obtained by sequence alignment by searching for Nr, Nt, Swissprot, Pfam, and KEGG public databases. TF analysis is extracted directly from the PlantTFDB database(http://planttfdb.cbi.pku.edu.cn/). All raw data and datasets were submitted to the China National Center for Bioinformation under the BioProject PRJCA056224.

Principal component analysis (PCA) was performed to reveal associations among treatments. Differential gene expression analysis was performed by gene counts using DESeq2, with genes satisfying |log2FC| ≥ 1 and *padj*-value < 0.05 considered differentially expressed genes (DEGs). Gene Ontology (GO) and Kyoto Encyclopedia of Genes and Genomes (KEGG) enrichment analyses were performed using clusterProfiler (v4.6.2), with a significance threshold of p < 0.05.

### 2.4 Metabolite extraction and untargeted metabolic analysis

Metabolomics samples were collected from the same plants and leaf position used for RNA-seq. Three independent biological replicates were performed for each group. Collected samples were immediately frozen in liquid nitrogen, and 80 mg leaves were weighed and extracted by directly adding 1 mL cold methanol/acetonitrile/H_2_O (2:2:1, v/v/v) containing internal standards to homogenized solution for metabolite extraction. The mixture was centrifuged for 20 min (14000 g, 4 °C). After filtering, 600 μL of the supernatants were transferred to autosampler vials for LC-MS/MS analysis. Analysis was performed using an UHPLC (1290 Infinity LC, Agilent Technologies) coupled to a quadrupole time-of-flight (AB Sciex TripleTOF 6600) in Shanghai Applied Protein Technology Co., Ltd. Raw data were converted to MzXML files using ProteoWizard, and then peak alignment, retention time correction and peak area extraction were performed using XCMS software. CAMERA (Collection of Algorithms of MEtabolite pRofile Annotation) was sued for annotation of isotopes and adducts. Compound identification of metabolites was performed by comparing of accuracy m/z value (<10 ppm), and MS/MS spectra with an in-house database established with available authentic standards.

After sum-normalization, the processed data were analyzed by R package, where it was subjected to multivariate data analysis, including Pareto-scaled principal component analysis (PCA) and orthogonal partial least-squares discriminant analysis (OPLS-DA). In this study, the variable importance in the projection (VIP) value of each variable in the OPLS-DA model was calculated to indicate its contribution to the classification. Student’s t test was applied to determine the significance of differences between two groups of independent samples. The value of VIP > 1, combined with fold change (FC) ≥ 1.2 or ≤ 0.83 and p value < 0.05 (Akarachantachote et al., 2014) were set as the filtering condition to choose metabolites with different abundance (DAM). The enrichment analyses and heatmap of differentially abundance metabolites (DAM) were obtained in R using the packages of ggplot2 and ComplexHeatmap, respectively.

### 2.5 Multi-omics integration and qRT-PCR analysis

Omics integration included: (1) DEGs and DAMs were mapped to KEGG (gene IDs and compound IDs) to identify concordant pathways. (2) Pairwise Pearson correlations were computed between DEG expression and DAM abundances; edges with |r| ≥ 0.6 and FDR < 0.05 were retained. (4) DIABLO (mixOmics) (Rohart et al., 2017) was used to identify multi-omics signatures discriminating genotype × treatment. Networks were visualized in Cytoscape.

Total RNAs were extracted from plant leaves of the same plot using TRIzol according to the manufacturer’s protocol, and three biological replicated were used. Total RNA was treated with gDNA Removal and used for the cDNA synthesis supermix kit (TransGen Biotech, Beijing, China). The qRT-PCR assays were performed using the SYBR Mix Kit (TransGen Biotech, Beijing, China) and were conducted on a Roche LightCycler 96 Sequence Detection System. The reference gene, *CdActin*, was used to normalize the expression levels of target genes. The expression levels were calculated using the 2^–ΔΔCt^ method. The qRT-PCR analysis was performed to validate the reliability of the RNA-seq data and analyze the expression level of essential genes.

### 2.6 Data analysis

Physiological and qRT-PCR data were analyzed in R (version 4.5.2) and/or Graph Prism 9. Data are presented as mean ± standard error (SE) of at least three independent biological replicates. Data were analyzed by one-way or two-way analysis of variance (ANOVA) followed by Fisher’s LSD test Differences were considered statistically significant at p < 0.05.

## 3 Results

### 3.1 Physiological characterization reveals enhanced drought tolerance in the *ems21S373204* mutant

**Figure 1:**
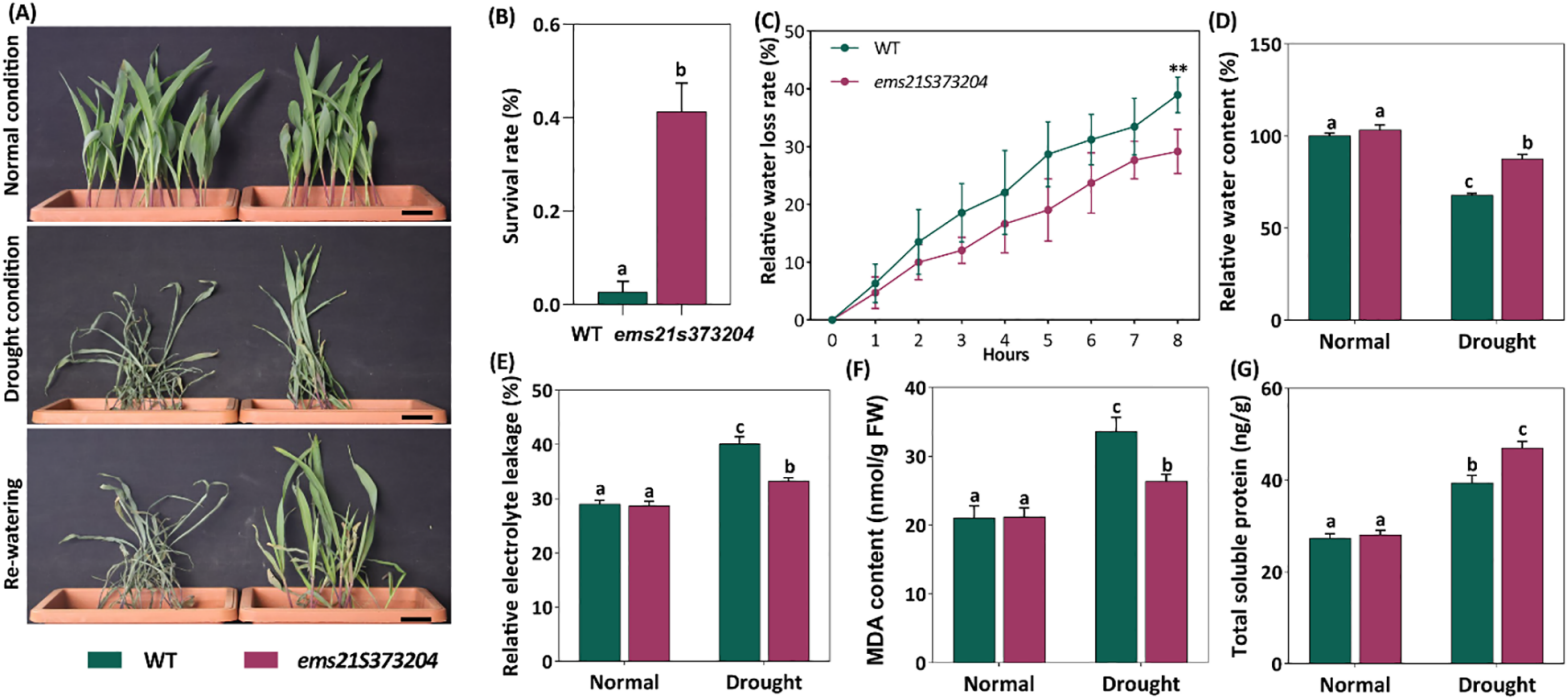
Phenotypic and physiological characterization of the WT and *ems21S373204* mutant under drought stress. **(A)** Representative phenotypes of WT and mutant plants grown under normal conditions, subjected to drought stress, and after re-watering. Scale bar, 1cm **(B)** Survival rates of plants recorded 3 days after re-watering. **(C)** Relative water loss rates of detached leaves measured over an 8-hour period. **(D–G)** Physiological indices measured under normal and drought conditions: **(D)** Relative water content (RWC); **(E)** Relative electrolyte leakage (REL), indicating cell membrane integrity; **(F)** Malondialdehyde (MDA) content, indicating the extent of lipid peroxidation; and **(G)** Total soluble protein content. Data represent means ± SD (n ≥ 3). Bars labeled with different letters differ significantly (*p* < 0.05, Fisher’s LSD). Asterisks indicate significant differences (**p < 0.01) based on Student’s *t*-test.

To evaluate the drought tolerance of the EMS-induced mutant, phenotypic and physiological traits was compared between WT and the *ems21S373204* mutant. Under normal conditions, no significant morphological differences were observed between the genotypes (**Figure 1A**). However, following drought treatment, severe leaf rolling and wilting were exhibited by the WT plants, whereas the mutant maintained a relatively good performance. Upon re-watering, the mutant plants recovered effectively, showing a significantly higher survival rate compared to the WT (**Figures 1A, B**).

To investigate the physiological basis of this phenotype difference, water status and membrane integrity under drought stress were assessed. The relative water loss rate of detached leaves was found to be consistently lower in the mutant than in the WT over an 8-hour period (**Figure 1C**). Consistently, under drought conditions, a higher RWC was maintained in the mutant leaves (**Figure 1D**). Furthermore, cell membrane stability was evaluated by measuring REL and MDA content. While drought stress induced increases in both REL and MDA levels across both genotypes, these increases were significantly mitigated in the *ems21S373204* mutant compared to the WT (**Figures 1E, F**). Additionally, the total soluble protein content, which potentially contributes to osmotic adjustment, was significantly upregulated in the mutant under stress conditions (**Figure 1G**). Taken together, the results demonstrate that the *ems21S373204* mutant displays enhanced drought tolerance, which is closely associated with enhanced water retention, improved membrane stability, and systematic adjustments at both transcriptional and metabolic levels.

### 3.2 Transcriptomic reprogramming and alteration of stress-responsive pathways in the *ems21S373204* mutant under drought stress

**Figure 2:**
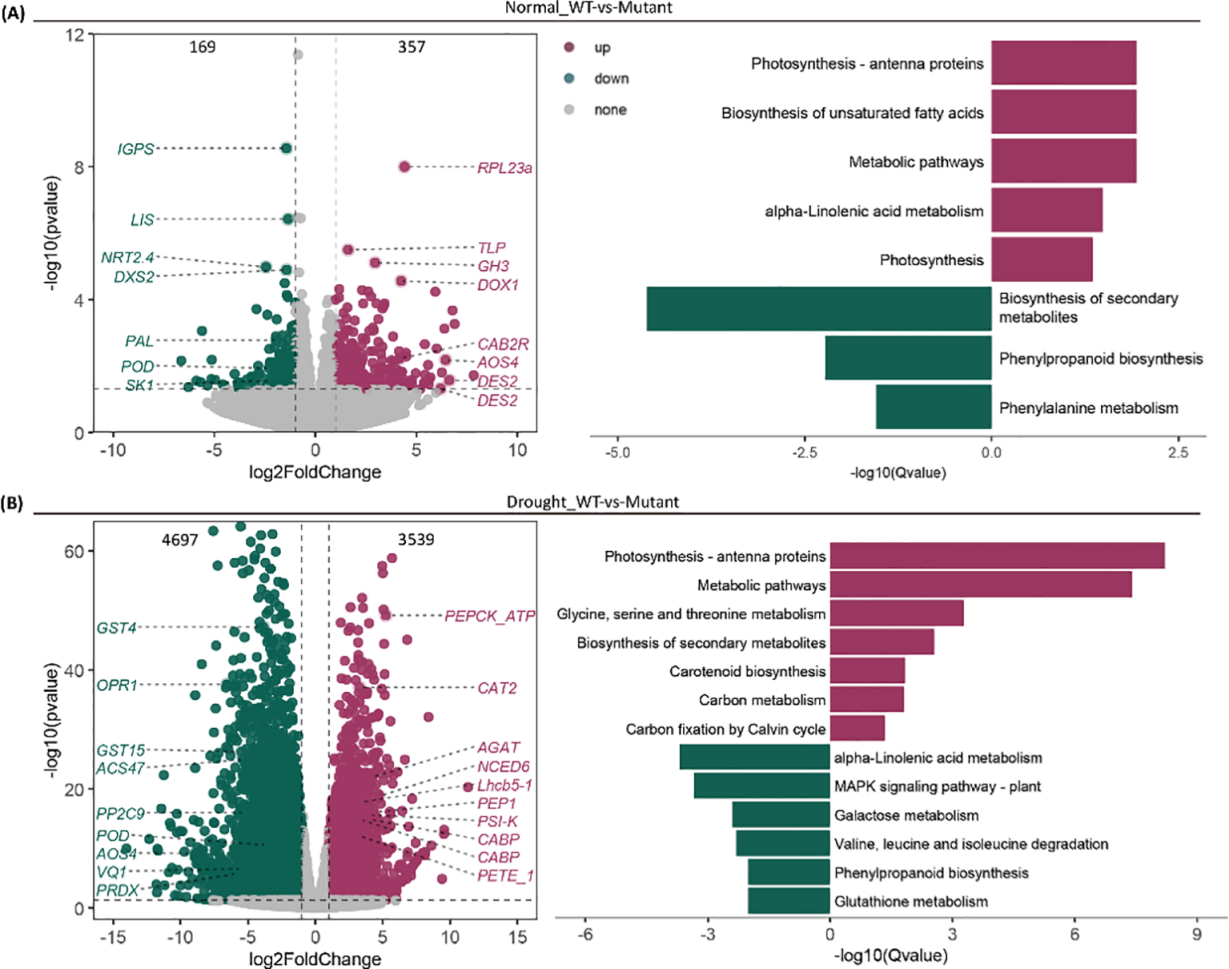
Comparative transcriptomic analysis of WT and *ems21S373204* mutant under normal and drought conditions. Volcano plots (left) and Bar chart (right) of KEGG pathway enrichment of differentially expressed genes (DEGs) between WT and mutant plants under **(A)** normal conditions and **(B)** drought stress. In the volcano plots, the x-axis represents the log2FoldChange (log2FC), and the y-axis represents the significance level (−log10pvalue). Upregulated and downregulated genes in the mutant compared to the WT are indicated by purple and green dots, respectively, while grey dots represent genes with no significant difference. The dashed vertical lines in volcano plots denote the threshold for significance (∣log2FC∣≥1 and *p* < 0.05). Key DEGs involved in stress responses and metabolism are labeled and the full names of key DEGs were presented in **Supplementary Table 1**. In the bar charts, the x-axis represents the significance of pathway enrichment (−log10Qvalue). Purple bars indicate pathways enriched with upregulated genes, and green bars indicate pathways enriched with downregulated genes.

To elucidate the molecular mechanisms underlying the enhanced drought tolerance of the *ems21S373204* mutant, comparative transcriptomic analyses were performed between the WT and the mutant under both normal and drought conditions. Under normal conditions, a relatively limited number of DEGs were identified, with 357 genes upregulated and 169 genes downregulated in the mutant compared to the WT (**Figure 2A**). KEGG pathway enrichment analysis showed that genes associated with photosynthesis-antenna proteins and unsaturated fatty acid biosynthesis were significantly upregulated in the mutant, indicating that the mutant might possesses a distinctive basal metabolic profile.

In contrast, drought stress induced extensive transcriptomic reprogramming. A total of 8, 236 DEGs were identified between the distinct genotypes under drought conditions, comprising 3, 539 upregulated and 4, 697 downregulated genes (**Figure 2B**). Notably, several key drought-responsive genes showed favorable expression patterns in the mutant. For instance, *NCED6*, a key gene involved in abscisic acid (ABA) biosynthesis, and *CAT2*, which encodes a catalase involved in ROS scavenging, were significantly upregulated. Meanwhile, protein phosphatase (*PP2C9*), a negative regulator of ABA signaling, was downregulated. Consistently, KEGG analysis under drought conditions indicated a sustained upregulation of photosynthesis-related pathways, carotenoid and carbon metabolism in the mutant. These transcriptomic alterations suggest that the *ems21S373204* plant activates a robust regulatory network involving enhanced ABA signaling and antioxidant defense to cope with water deficit. The result of qRT-PCR on *CAT2, NCED6* and *PP2C9* gene (**Supplementary Figure 1**), verified the key pathway.

### 3.3 Metabolic profiling reveals distinct reconfiguration of nucleotide and amino acid metabolism in the *ems21S373204* mutant

**Figure 3:**
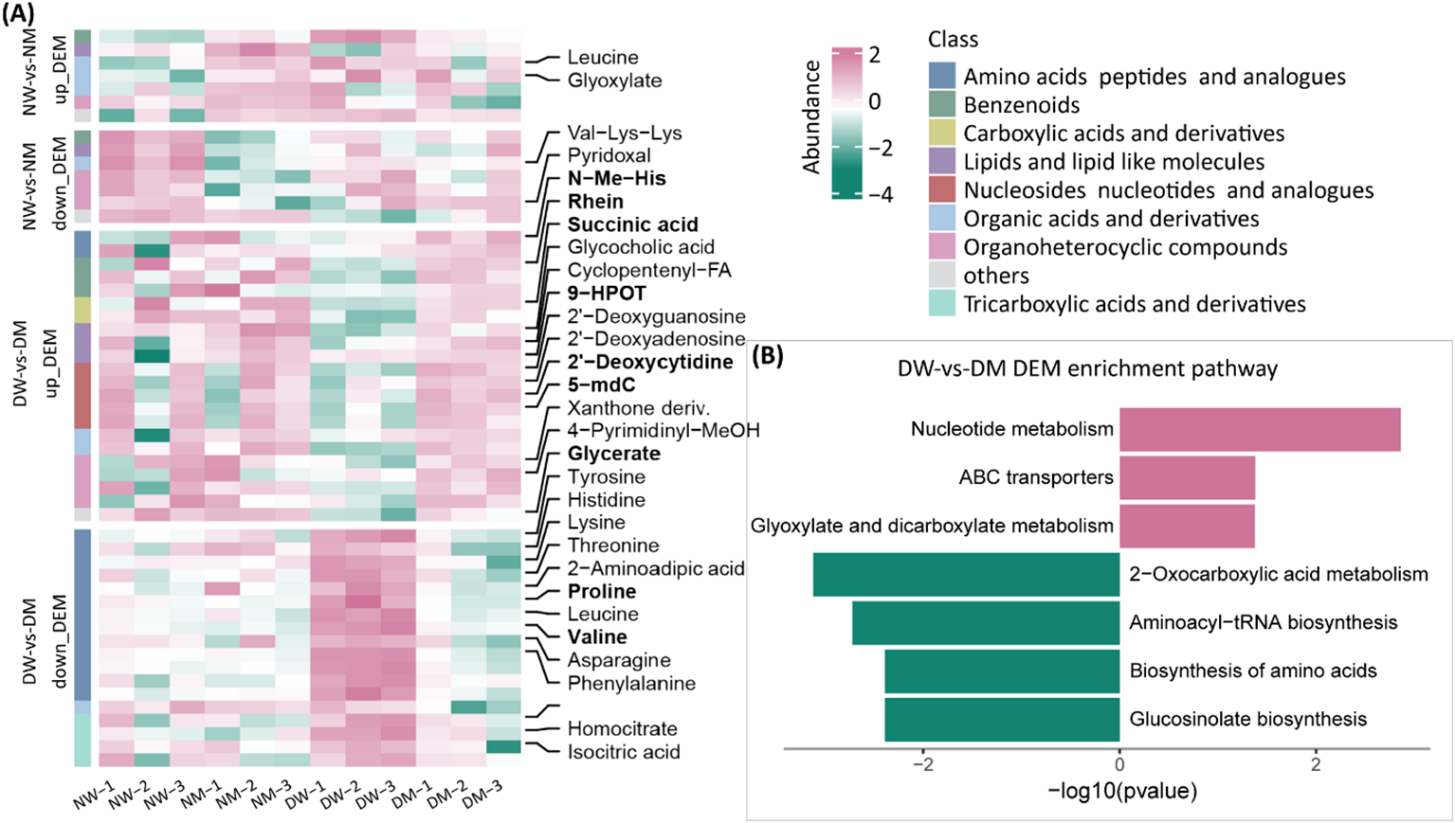
Comparative metabolomic analysis of WT and *ems21S373204* mutant under normal and drought conditions. **(A)** Heatmap visualization of differential abundance of metabolites (DAMs). The color scale represents the relative abundance of metabolites (normalized Z-score), with pink indicating upregulation and green indicating downregulation. Columns represent biological replica tes (n ≥ 3) for Normal-WT (NW), Normal-Mutant (NM), Drought-WT (DW), and Drought-Mutant (DM). Key metabolites are labeled and the full names of key DAMs were presented in **Supplementary Table 1**. **(B)** KEGG pathway enrichment analysis of DAMs identified between WT and mutant under drought conditions (DW-vs-DM). Purple bars represent pathways enriched with upregulated metabolites, while green bars represent pathways enriched with downregulated metabolites. The x-axis represents the significance of enrichment (−log10pvalue).

To further explore the metabolic signatures associated with the drought-tolerant phenotype, an untargeted metabolomics analysis was performed on leaves from WT and mutant plants under both normal and drought conditions. Differential abundance analysis of metabolites (DAMs) revealed distinctive metabolic profiles between the two genotypes, particularly under water deficit (**Figure 3A**). Under normal conditions, relatively minor differences were observed; however, specific metabolites such as glyoxylate were upregulated, while succinic acid was downregulated in the mutant compared to the WT.

Under drought stress, a substantial metabolic reconfiguration was identified. Notably, a cluster of nucleotide-related metabolites, including 2’-deoxycytidine and 5-mdC, showed significantly higher abundance in the mutant than in the WT. Consistently, nucleotide metabolism was the most significantly enriched pathway (**Figure 3B**), supporting altered nucleotide homeostasis in the mutant under drought stress. Furthermore, a significant upregulation of glycerate, a pivotal intermediate in the photorespiratory pathway, was observed in the mutant (**Figure 3A**). This aligns with the pathway enrichment of glyoxylate and dicarboxylate metabolism (**Figure 3B**). The accumulation of glycerate implies a robust maintenance of photorespiratory flux, which likely serves to dissipate excess excitation energy and recycle carbon, thereby protecting the photosynthetic apparatus from photoinhibition.

Conversely, a marked downregulation of free amino acids (e.g., proline, valine, leucine) was observed in the mutant relative to the WT under drought conditions, accompanied by the suppression of biosynthesis of amino acids (**Figures 3A, B**). The lower accumulation of free amino acids suggests attenuated stress-induced proteolysis, whereas the elevated levels in the WT likely reflect severe protein degradation triggered by cellular damage. Collectively, these metabolic shifts indicate that the *ems21S373204* mutant coordinates nucleotide protection, photorespiratory adjustment, and protein homeostasis to survive water deficit.

### 3.4 Integrated analysis reveals a coordinated regulatory network connecting gene expression and metabolic flux

**Figure 4:**
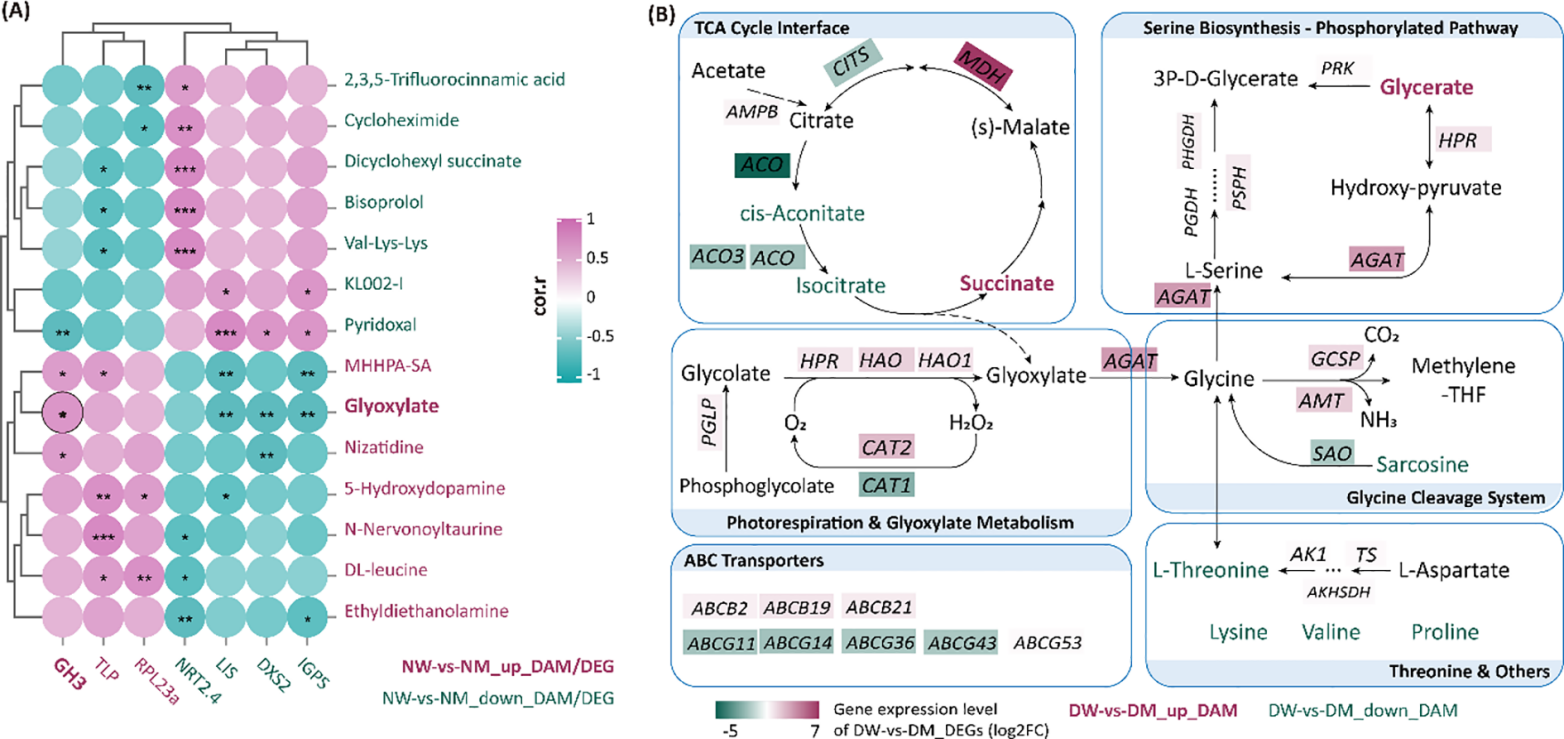
Integrated transcriptomic and metabolomic analysis of the *ems21S373204* mutant. **(A)** Correlation heatmap of differentially expressed genes (DEGs) and differential abundance of metabolites (DAMs) identified under normal conditions. The color scale indicates the Spearman correlation coefficient. **(B)** Pathway-based integration of DEGs and DAMs under drought conditions. Key metabolic and regulatory modules, including the TCA cycle interface (the linkage points connecting the TCA cycle with photorespiration and glyoxylate metabolism), Serine Biosynthesis - Phosphorylated Pathway, Photorespiration & Glyoxylate Metabolism, Glycine Cleavage System, ABC transporters, Threonine and Others, are schematically represented. Rectangular boxes represent genes, and text labels represent metabolites. Solid arrows represent direct metabolic steps, and dashed arrows represent multi-step or indirect connections. The full names of key genes and metabolites were presented in **Supplementary Table 1**. The symbols *, **, and *** indicate statistical significance levels: P < 0.05, P < 0.01, and P < 0.001, respectively. In panel **(A)**, they refer to the significance of Spearman correlation coefficients.

To explore the regulatory relationships between the transcriptomic and metabolomic alterations, a multi-omics integration analysis was performed. Under normal conditions, a correlation analysis between DEGs and DAMs revealed that the accumulation of glyoxylate was significantly positively correlated with the expression of specific upregulated genes, such as *GH3* (an auxin-responsive gene), while showing a negative correlation with downregulated genes like *LIS* and *DXS2* (**Figure 4A**). This suggests a pre-existing coordination between glyoxylate metabolism and stress-related gene expression in the mutant even prior to stress exposure.

Under drought conditions, both DEGs and DAMs was mapped onto key metabolic pathways, including ABC transporters, Glycine, serine and threonine metabolism, Phenylpropanoid biosynthesis, and Glyoxylate and dicarboxylate metabolism (**Supplementary Figure 2**; **Figure 4B**). Notably, several critical regulatory genes were significantly differentially expressed, including *THAUMATIN-LIKE PROTEIN* (*TLP*), *INDOLE-3-GLYCEROL PHOSPHATE SYNTHASE* (*IGPS*), and genes involved in the MHHPA-SA pathway, which were closely associated with stress defense and metabolic homeostasis. In the TCA cycle interface, the downregulation of *ACO* (encoding aconitase) coincided with a marked depletion of its catalytic products, cis-aconitate and isocitrate. Conversely, the upregulation of *MDH* (malate dehydrogenase) was accompanied by the accumulation of succinate. In the photorespiration and glyoxylate metabolism modules, a distinct isoform switch from *CAT1* (downregulated) to *CAT2* (upregulated) was observed. Furthermore, the upregulation of genes encoding key photorespiratory enzymes, including *AGAT* encoding alanine-glyoxylate transaminase and *HAO* encoding (S)-2-hydroxy-acid oxidase, paralleled the significant accumulation of glycerate.

Notably, a divergence in gene expression was observed within the ABC transporter superfamily (**Figure 4B**). Members of the *ABCG* subfamily (e.g., *ABCG11*, *ABCG36*) were consistently downregulated. In contrast, members of the *ABCB* subfamily, key regulators of auxin transport, including *ABCB2* and *ABCB19*, were significantly upregulated. Their upregulation aligns with the elevated expression of the auxin-responsive gene *GH3* identified in the correlation analysis, suggesting a modulated auxin transport system in the mutant under stress. Consistent with these changes, the altered expression of TLP, IGPS, and genes in the MHHPA-SA pathway further reinforced the activation of stress-responsive regulatory networks, which coordinated with downstream metabolic shifts. Additionally, the depletion of free amino acids, such as valine and proline, was mapped alongside alterations in their respective biosynthetic or degradation pathways. These integrated results indicate that the metabolic shifts observed in the *ems21S373204* mutant are closely correlated with corresponding genome-wide transcriptional changes, supporting a coordinated transcript-metabolite regulatory pattern in response to drought stress.

## 4 Discussion

The primary objective of this study was to elucidate the systemic mechanisms underlying the enhanced drought tolerance exhibited by the EMS-induced maize mutant *ems21S373204*. While previous studies have successfully used EMS mutagenesis to identify drought-resistant germplasm in maize (Cho et al., 2020; Dhannoon et al., 2020) and wheat (Le Roux et al., 2021; Zahra et al., 2022), most have focused on individual physiological traits or single-gene functional characterization. Our study provides a more integrated perspective, showing that the *ems21S373204* mutant displays a multi-faceted physiological and metabolic response associated with enhanced ABA signaling, improved antioxidant capacity, and coordinated metabolic rearrangement toward photorespiratory carbon recycling. Although our findings reveal key mechanisms contributing to drought tolerance in this mutant, we acknowledge that a longer recovery period (7–14 days) would better reflect long-term stress resilience and represents a limitation to be addressed in future investigations. Overall, these results indicate that *ems21S373204* maintains stable cellular homeostasis under drought stress, supporting its value as a promising genetic resource for drought resistance breeding.

### 4.1 Distinct physiological homeostasis maintained by ABA signaling and protein stability

The enhanced physiological performance of the *ems21S373204* mutant under water deficit condition is fundamentally supported by a distinct regulatory mechanism regarding ROS scavenging and protein homeostasis. Our results showed that the mutant exhibited significantly higher RWC and lower MDA levels compared to the WT (**Figure 1**). This aligns with findings by Dhannoon et al. (2020) and Cho et al. (2020), where drought-tolerant maize mutants demonstrated reduced oxidative damage. Although this study did not determine chlorophyll content or measure catalase and other antioxidant enzyme activities (Li et al., 2021; Tang et al., 2023; Zhao et al., 2025), our transcriptomic data revealed significant upregulation of the catalase gene CAT2 and the ABA biosynthesis gene NCED6 in the mutant under drought stress. Future enzyme activity assays will be needed to functionally validate these transcriptional changes. Unlike the recently characterized *ZmDnaJ*-*ZmNCED6* module, where the ZmDnaJ chaperone positively regulates drought tolerance by stabilizing ZmNCED6 protein, the *ems21S373204* mutant simultaneously upregulates *NCED6* (biosynthesis) and down-regulates *PP2C9* (a negative regulator of ABA signaling) (**Figure 2**). This indicates that the mutant employs a transcriptional synergistic strategy for ABA signaling, rather than relying solely on protein stability.

Furthermore, prior EMS-based studies in wheat (Le Roux et al., 2021; Zahra et al., 2022) and maize (Cho et al., 2020) has identified the accumulation of free amino acids (e.g., proline) as a key osmoprotection mechanism. However, our metabolomic analysis revealed markedly reduced levels of free amino acids but higher total soluble protein content in the mutant compared to the WT (**Figures 1G**, **3**). This discrepancy implies that the elevated abundance of free amino acid in the WT likely attributable to stress-induced proteolysis. Conversely, the *ems21S373204* mutant maintained more stable cellular protein levels under drought stress compared with the wild type, with less pronounced protein degradation.

### 4.2 Metabolic reconfiguration: the pivotal roles of nucleotide turnover and photorespiration

Beyond physiological traits, our multi-omics integration reveals a distinctive metabolic reconfiguration strategy centered on photorespiration and nucleotide turnover, distinguishing *ems21S373204* from other characterized mutants. Recent studies have demonstrated that *ZmATG18a*-mediated autophagy confers drought tolerance by recycling damaged cellular components (Z. Wang et al., 2025). While autophagy serves as a survival mechanism through the recycling of damaged components, our data indicate that the *ems21S373204* mutant employs a damage prevention strategy via the photorespiratory glycolate pathway. Our integrated analysis identified a coordinated upregulation of *AGAT* and *HPR* genes with the accumulation of the metabolite glycerate (**Figure 4**). Unlike C4 photosynthesis models where photorespiration is often considered negligible or wasteful (Wingler et al., 1999), our findings suggest that under drought-induced stomatal closure, the mutant actively engages this pathway (Ünlüsoy et al., 2023). This likely functions as a crucial energy dissipation mechanism to consume excess reducing power (NADPH/ATP), thereby protecting photosystems from photoinhibition. Additionally, the specific enrichment of nucleotide metabolism (**Figure 3**) and the upregulation of DNA repair-related metabolites (e.g., deoxycytidine), thereby facilitating rapid recovery upon re-watering (**Figure 1A**). Collectively, these findings delineate a novel adaptive strategy in *ems21S373204*: instead of undergoing a passive metabolic shutdown or engaging in extensive autophagy, the mutant actively redirects carbon flux towards photorespiration and prioritizes genomic integrity, ensuring survival without compromising recovery potential.

Based on the integrated phenotypic, transcriptomic, and metabolomic datasets, a model has been proposed to describe the drought tolerance mechanism in the *ems21S373204* mutant. Upon detection of water deficit signals, the mutant induces the expression of *NCED6* while suppressing the negative regulator *PP2C9*, leading to enhanced ABA signaling. This initial signal triggers a cascade of downstream events: (1) the transcriptional activation of antioxidant genes (e.g., *CAT2*) to precisely control ROS levels and prevent membrane lipid peroxidation; (2) the redirection of metabolic flux involved transcriptional upregulation of genes encoding ABCB transporters potentially related to auxin transport and activation of the photorespiratory module (AGAT, HPR, and glycerate) to dissipate excess energy and recycle carbon; and (3) the maintenance of protein homeostasis, evidenced by reduced proteolysis and stable soluble protein levels. These molecular and metabolic adjustments collectively mitigate cellular damage, preserve turgor pressure, and ultimately result in the “stay-green” and high-survival phenotype observed.

In conclusion, this study systematically characterized the *ems21S373204* mutant and revealed that its enhanced drought tolerance is derived from a coordinated network of enhanced ABA signaling, robust antioxidant defense, and adaptive metabolic reconfiguration, particularly in photorespiration and protein homeostasis. These findings provide valuable insights into the complex regulatory networks governing drought adaptation in maize and position the *ems21S373204* mutant as a promising germplasm for breeding climate-resilient varieties. However, a limitation of the current study is that the specific causal single-nucleotide polymorphism (SNP) responsible for these pleiotropic effects has not yet been pinpointed, as EMS mutagenesis generates genome-wide variations. Future research will focus on identifying the mutation site through map-based cloning or SNP analysis and validating its function via CRISPR/Cas9 gene editing. Additionally, further field trials under diverse environmental conditions are warranted to assess the yield stability of this mutant, bridging the gap between basic research and agronomic application.

## Data Availability

The datasets presented in this study can be found in online repositories. The names of the repository/repositories and accession number(s) can be found below: https://ngdc.cncb.ac.cn/gsa/browse/CRA038442.
