# Supplementary material for: A coordinated network of ABA signaling, photorespiratory and proteostasis in EMS-induced maize mutant with enhanced drought resistance
Source: Front Plant Sci. 2026 Jun 12;17:1828125. doi: 10.3389/fpls.2026.1828125 (PMC13305815; doi:10.3389/fpls.2026.1828125)
Supplement: Supplementary file 1 [file SupplementaryFile1.docx]

Supplementary Material

# Supplementary Tables

**Supplementary Table 1.** List of full names or annotation information of genes and metabolites in this study.

| **Names (Abbr.)** | **Full name**  **(or annotation info.)** | **Names (Abbr.)** | **Full name**  **(or annotation info.)** |
| --- | --- | --- | --- |
| ACO | Aconitate hydratase | OPR1 | 12-oxo-phytodienoic acid reductase1 |
| ACS47 | 1-aminocyclopropane-1-carboxylate synthase | PAL | Phenylalanine ammonia-lyase |
| AGAT | alanine--glyoxylate transaminase | PEP1 | phosphoenolpyruvate carboxylase 1 |
| AK1 | aspartate kinase | PEPCK_ATP | phosphoenolpyruvate carboxykinase (ATP) |
| AKHSDH | aspartokinase/homoserine dehydrogenase | PETE_1 | Plastocyanin |
| AMT | Aminomethyltransferase | PGDH | D-3-phosphoglycerate dehydrogenase |
| AOS4 | allene-oxide synthase4 | PGLP | phosphoglycolate phosphatase |
| CAB2R | chlorophyll a-b binding protein of LHCII type 1 | PHGDH | D-3-phosphoglycerate dehydrogenase |
| CABP | Chlorophyll a-b binding protein, chloroplastic | POD | Peroxidase |
| CAT1 | catalase 1 | PP2C9 | probable protein phosphatase 2C 8 |
| CAT2 | catalase 2 | PRDX | Peroxiredoxin |
| DES2 | fatty acid desaturase DES2 | PRK/URK | Phosphoribulokinase/uridine kinase domain-containing protein |
| DOX1 | Alpha-dioxygenase 1 | PSI-K | Photosystem I reaction center subunit psaK, chloroplastic |
| DXS2 | deoxy xylulose synthase 2 | PSPH | phosphoserine phosphatase |
| GCSP | Glycine cleavage system P protein | RPL23a | 60S ribosomal protein L23a |
| GH3 | Glycosyl hydrolase family protein | SAO | Putative sarcosine oxidase |
| GST15 | glutathione transferase 15 | SK1 | shikimate kinase |
| GST4 | glutathione transferase 15 | TLP | Thaumatin-like protein |
| HAO | (S)-2-hydroxy-acid oxidase | TS | threonine synthase |
| HPR | glycerate dehydrogenase | VQ1 | VQ domain-containing protein |
| IGPS | indole-3-glycerol-phosphate synthase | N-Me-His | N-Methyl-L-histidine |
| Lhcb5-1 | Chlorophyll a-b binding protein, chloroplastic | Cyclopentenyl-FA | 3-Cyclopentene-1-octanoic acid, 2-(3-hydroxy-1-penten-1-yl) |
| LIS | S-linalool synthase | 9-HPOT | 9-Hydroperoxy-10E,12Z,15Z-octadecatrienoic acid |
| MDH | malate dehydrogenase | 5-mdC | 5-Methyl-2'-deoxycytidine |
| NCED6 | Nine-cis-epoxycarotenoid dioxygenase6 | Xanthone deriv. | 3a,6-Dihydroxy-9-(3-hydroxy-3-methylbutyl)-4-methoxy-3a,12a-dihydro-5H-furo[3',2':4,5]furo[3,2-b]xanthen-5-one |
| NRT2.4 | High-affinity nitrate transporter 2.4 | 4-Pyrimidinyl-MeOH | 4-Pyrimidinylmethanol |

# Supplementary Figures


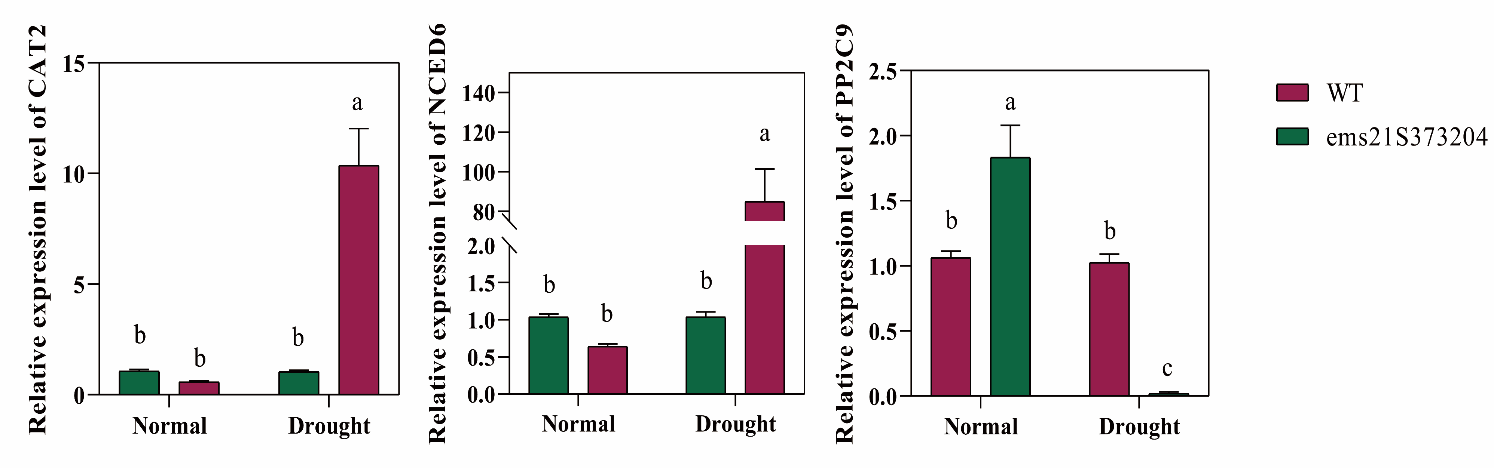


**Supplementary Figure 1.** Result of qRT-PCR analysis on *CAT2*, *NCED6* and *PP2C9* gene


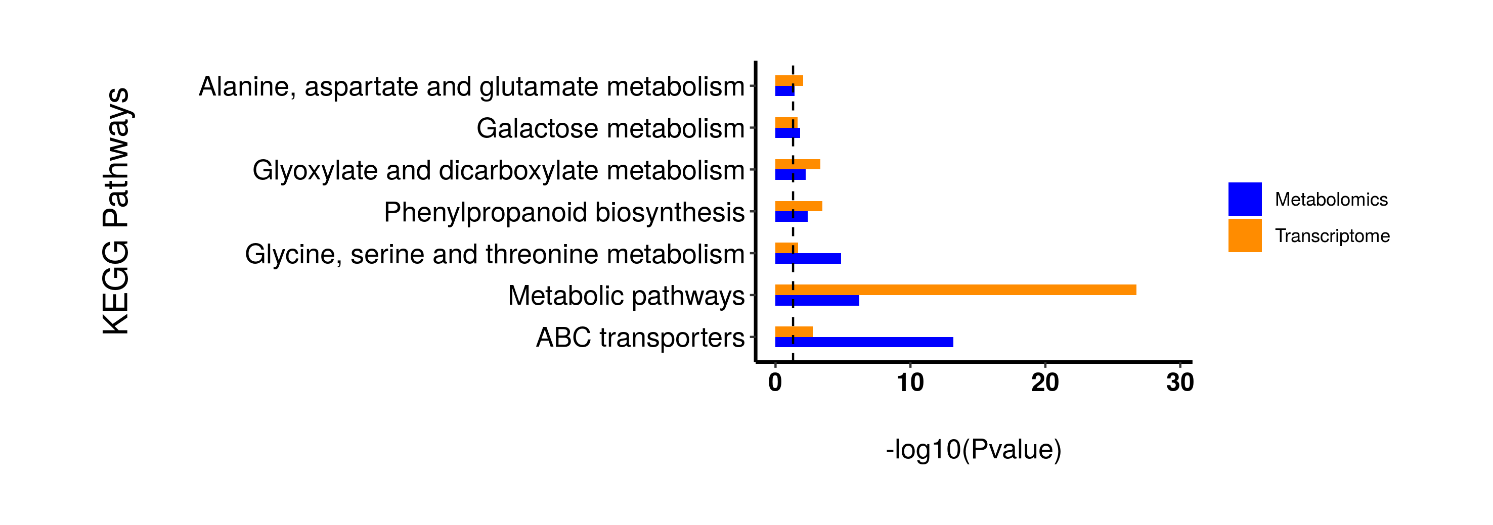


**Supplementary Figure 2.** Enriched analysis of both DEGs and DAMs mapped onto key KEGG metabolic pathways.
